# Suspected medullary washout leading to severe polyuria following delayed cerebral ischemia: a case report

**DOI:** 10.1186/s12882-023-03281-4

**Published:** 2023-09-01

**Authors:** Pape-Mamadou Sene, Ahmad Gebai, Tal Kopel, Jean-François Cailhier, Dominique Lafrance, Jean-Maxime Côté

**Affiliations:** 1 Division of Nephrology, Department of Medicine, Centre Hospitalier de l’Université de Montréal, Montréal, Canada; 2 Centre de recherche du Centre Hospitalier de l’Université de Montréal, Montréal, Canada; 3 Division of Intensive Care, Department of Medicine, Centre Hospitalier de l’Université de Montréal, Montréal, Canada

**Keywords:** Cerebral salt wasting, Subarachnoid hemorrhage, Polyuria, Dysnatremia, ADH, Urine concentration defect, Delayed cerebral ischemia, Case report

## Abstract

**Background:**

Delayed cerebral ischemia is a clinical entity commonly encountered in patients presenting with acute neurological injury and is often complicated by dysnatremias, such as the cerebral salt wasting syndrome. In this case report, we described an exceptional case of polyuria attributed to an initial cerebral salt wasting phenomenon and iatrogenic-induced medullary washout.

**Case presentation:**

A 53-year-old woman was admitted to our hospital for the management of a Modified Fisher scale grade 4 subarachnoid hemorrhage due to a ruptured posterior communicating aneurysm. She was initially managed with coil embolization and external ventricular drain due to secondary hydrocephalus. Throughout the course of her hospitalization, she developed severe polyuria reaching up to 40L per day. To keep up with the excessive urinary losses and maintain appropriate cerebral perfusion, fluid replacement therapy was adjusted every hour, reaching up to 1.3 L of crystalloid per hour in addition to aminergic support. An initial diagnosis of partial diabetes insipidus, followed by a cerebral salt wasting syndrome was suspected. While the urine output continued to increase, her serum urea concentration progressively decreased to a point of almost being undetectable on day 9. At that time, the presence of an interstitial medulla washout was hypothesized. Various pharmacological and non-pharmacological interventions were progressively introduced to regain normal renal homeostasis, including non-steroidal anti-inflammatory drugs, fludrocortisone, oral urea and high-protein intake. Medications were progressively weaned, and the patient was successfully discharged from the ICU.

**Conclusions:**

Cerebral salt wasting should be considered in the initial differential diagnosis of a patient presenting with polyuria in the context of acute neurological injury. Early recognition of this entity is critical to quickly implement proper management. However, as shown in this case report, the concomitance of delayed cerebral ischemia may complexify that management.

## Background

Subarachnoid hemorrhage (SAH) typically follows a biphasic course with a phase of early brain injury followed by delayed cerebral ischemia (DCI) in 20–30% of patients. Clinically, DCI is defined as new focal neurological deficit or decrease in modified Glasgow Coma Score (GCS) or abbreviated National Institute of Health Stroke Scale (NIHSS) in the absence of other causes that could contribute to clinical neurologic deterioration such as fever or electrolyte abnormalities [1–3]. Numerous mechanisms have been implicated in the pathogenesis of DCI which include vasospasm, neuroinflammation, microthromboembolism, and alteration in cerebral autoregulation. The management of DCI must address insults contributing to secondary brain injury, such as dysnatremia or hypovolemia [4, 5]. Dysnatremias are common in patients presenting with subarachnoid hemorrhage and other brain injuries, affecting up to 50% of hospitalized patients with acute neurological disorders [6]. Common causes of hyponatremia in patients presenting with SAH are the syndrome of inappropriate secretion of antidiuretic hormone (SIADH), leading to free water retention despite euvolemia; and cerebral salt wasting syndrome (CSW) leading to net negative sodium balance in the setting of ongoing hypovolemia [6, 7]. We describe an unusual case in which maintenance of euvolemia in a patient with high-grade SAH and severe vasospasm led to secondary alteration of the normal urine concentration physiology and ultimately, to an exceptional urine output of more than 40 L per day.

To the best of our knowledge, such a severe complication in the management of SAH has never been previously reported. This provides a good opportunity to revisit important principles of renal physiology.

## Case presentation

A 53-year-old woman, with a history of hypertension and depression, was transferred from a referring hospital with a presentation of sudden headache and loss of consciousness with an initial Glasgow Coma Scale (GSW) score between 4 and 6, requiring urgent intubation. The initial cerebral computed tomography (CT) scan revealed a Modified Fisher scale grade 4 SAH with moderate intraventricular hemorrhage and early signs suggestive of hydrocephalus. Upon arrival, a cerebrospinal fluid (CSF) diversion with an external ventricular drain was performed due to the initial altered level of consciousness with new-onset hydrocephalus. Later the same day, she successfully underwent balloon-assisted coil embolization of a ruptured 6 mm posterior communicating artery aneurysm, with no immediate angiographic evidence of complications. Prophylactic nimodipine was initiated and the patient underwent serial transcranial doppler ultrasonography every 48 h as recommended for the prevention of DCI. Two days later, following a rapid improvement of her neurological status, the patient was successfully extubated.

**Fig. 1:**
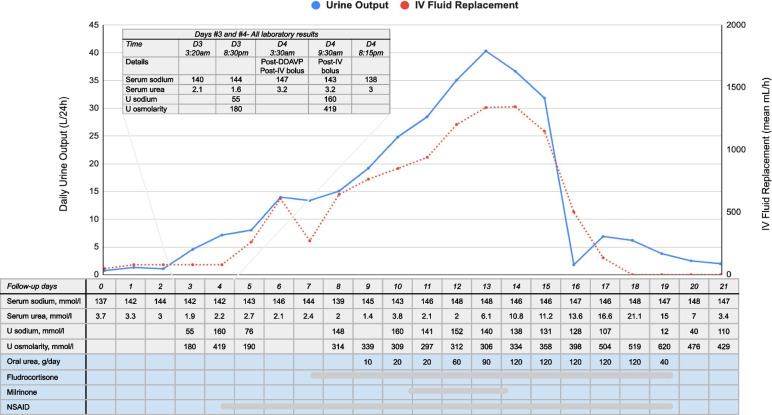
Daily urine output, intravenous fluid replacement volume and median laboratory values during the entire ICU stay. All available laboratory values from day 3 to day 4 are also presented. On day 4 – 3:30am, intravenous fluids included 0.9% NaCl and Ringer’s Lactate

On the third day of ICU admission, the urine output started to progressively increase reaching 4 L in a 24-h period, and was initially compensated with administration of 0.9% NaCl IV fluid. Later that day, she presented with a sudden decrease in consciousness, new-onset left ptosis, and lip drooping. An urgent CT scan and CT angiogram showed moderate vasospasm at the level of the anterior cerebral arteries and the posterior right cerebral artery. The patient’s DCI was therefore managed by IV crystalloid fluids to compensate for the increased urine output (see Day #3 and #4, Fig. 1). In the face of diagnostic uncertainty, a trial of 2 $\mu$ g of IV desmopressin to rule out central diabetes insipidus only showed mitigated results. The urine osmolality effectively increased from 180 to 419 mmol/L (following desmopressin and concomitant crystalloid administration) suggesting initially a central diabetes insipidus (two times increase in osmolality and > 300 mmol/L). However, despite desmopressin, polyuria persisted reaching up to 2L in the next 3 h of administration, making the clinical presentation in favor of a mixed component of partial central diabetes insipidus and an ongoing cerebral salt wasting. No hypotension episode was reported but the patient had a low central venous pressure (CVP) at that time and a fluctuating neurologic status partially responding to IV fluid administration. Then, despite the absence of associated hyponatremia or clear hypovolemia, the polyuria was attributed to an ongoing cerebral salt wasting phenomenon and was therefore compensated with large volume of isotonic fluid replacement. To maintain the desired cerebral perfusion pressure to treat the active DCI, a slightly positive fluid balance was achieved and aminergic support was initiated to reach a mean arterial pressure of ~ 115 mm Hg. Despite this treatment, her neurologic status fluctuated greatly over the next 48 h. A cerebral angiography showed ongoing severe distal vasospasm. Intra-arterial milrinone was locally injected, and an intravenous perfusion of milrinone was subsequently initiated.

On the following days, as shown in Fig. 1, the patient’s polyuria worsened progressively, reaching up to 40 L on day 13. To keep up with the excessive urinary losses, the patient’s fluid replacement therapy had to be adjusted every hour, reaching up to 1.3 L of crystalloid per hour, using Ringer ‘s lactate and 0.9% NaCl in combination to minimize hyperchloremia and electrolyte abnormalities. The patient demonstrated fluctuating neurological deteriorations in the form of diplopia and altered mental status when she was in negative fluid balance, limiting the capacity to reduce the cerebral perfusion pressure targeted. Additionally, her serum urea concentration progressively decreased to nearly indetectable levels on day 9. At that time, renal medullary washout induced by severe polyuria was hypothesized.

As shown in Fig. 1, various interventions were progressively initiated: 1) to reduce the glomerular filtration rate with high doses of non-steroidal anti-inflammatory drugs (NSAID)(naproxen up to 500 mg thrice daily followed by indomethacin 100 mg thrice daily); 2) to increase the distal sodium reabsorption with fludrocortisone (0.4 mg twice daily) and 3) aiming to re-normalize the renal medulla gradient with oral urea administration (30 g four times a day) and supra-physiologic protein intake (high-protein meals with oral beneprotein). Eventually, as the medullary concentration gradient was reconstituted and the targeted arterial pressure reduced, the urine output gradually decreased to the physiological range at day 16. Medications were progressively weaned on the following days until no intravenous fluid was required to maintain euvolemia (day 18). The patient was successfully discharged from the ICU at day 21 with no signs of DCI or cerebral infarction on a later MRI and was discharged at home with physical rehabilitation services.

## Discussion and conclusions

**Fig. 2:**
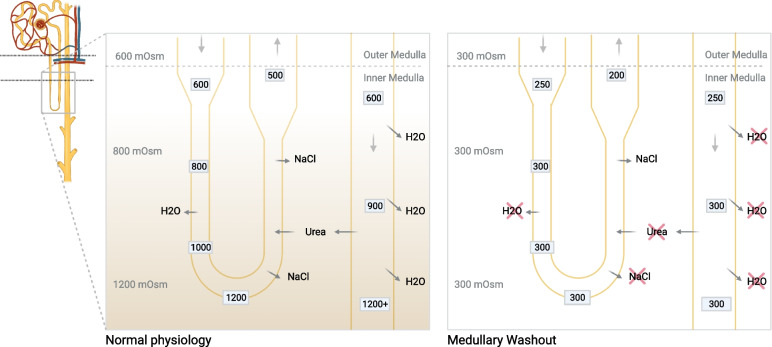
Gradient medullary concentration conceptual mechanism. Normal physiology: NaCl is actively reabsorbed across the thick ascending limb by the NKCC2 cotransporter. Water (H_2_O) is reabsorbed across the descending limb segment by AQP_1_ water channels. Water is reabsorbed across the collecting duct by AQP_2_ on the apical membrane in the presence of ADH, and AQP_2,3,4_ on the basolateral membrane. Urea (and other osmoles) are concentrated within the collecting duct lumen (by water reabsorption) until it reaches the terminal inner medulla segment, where urea is reabsorbed by the urea transporters (UT_A1_, UT_A3_). The inner medulla contains several urea recycling pathways that constantly contribute to maintain its high interstitial urea concentration (not show). Medullary Washout: In that absence of high interstitial urea concentration, the passive reabsorption of solutes and water despite the presence of ADH is limited, leading to high volume of iso-osmolar urine

We present an unusual case of a 53-year-old woman with subarachnoid hemorrhage, followed by complex and severe urine concentration abnormalities, implicating several physiology concepts (Fig. 2). The reasons for this polyuria were multifactorial and changed over the course of the ICU stay. The patient initially developed polyuria without remarkable abnormalities in her serum sodium concentrations. Initially, the diagnosis of partial central diabetes insipidus was retained as the urine osmolality increased and serum sodium concentration decreased following a trial of desmopressin. Then, to explain the inappropriate and persistent polyuria, a subsequent diagnosis of CSW was proposed despite the absence of hyponatremia, as all other typical characteristics were present: high and inappropriate urine output, high urine sodium concentration, high urine osmolality and relative signs of hypovolemia defined by low CVP and volume-dependent fluctuations of the neurologic status [8, 9]. Typically, the concomitant volume-sensitive ADH secretion leads to hyponatremia in classic CSW presentation, but we believe that the rapid IV fluid replacement set early during presentation could have been a confounding factor in this case, by inhibiting its relative secretion as the patient did not achieve the state of severe extracellular volume depletion. Instead, she was instead in a state of relative hypervolemia to maintain adequate cerebral perfusion.

CSW is a complex syndrome involving the brain-kidney axis and mainly characterized by extracellular volume depletion due to excessive sodium excretion in the context of intracranial pathologies such as SAH, stroke, or brain surgery. This entity is transitory after the onset of the neurological insult. The incidence of (classic) CSW in patients admitted to the intensive care unit for acute neurological injuries is approximately 11% [10]. Despite the clear association between CSW and neurological injuries, the pathophysiology leading to that loss of sodium and water homeostasis dysregulation is still debated, but interactions between the autonomic nervous system and humoral factors (natriuretic peptides [ANP, BNP] and ADH) are suspected. Following neurological injuries, there is increased secretion of ANP and BNP mostly from neurons surrounding the third ventricle in the anterolateral hypothalamic region. These natriuretic peptides take advantage of the disrupted blood–brain barrier to gain access to the systemic circulation and fulfill their physiological effects. Some authors also hypothesized that noradrenergic discharge triggers the production of BNP from the heart ventricles themselves (neuro-cardiac axis) [11]. These elevated natriuretic peptides contribute to the increased sodium excretion and urine volume by directly inhibiting tubular NaCl reabsorption, by limiting the intramedullary collecting-duct sodium absorption and by suppressing the release of renin by the juxtaglomerular cells and aldosterone by the adrenal gland despite volume depletion [12, 13].

Acute extracellular fluid depletion is the main feature of CSW. Thus, initial management should focus on the replacement of solute and water loss, usually with isotonic or hypertonic saline, to restore fluid and sodium balance. Since SAH is the most common cause of CSW and may be accompanied by volume-sensitive vasospasm, the risk of progressive hypovolemia should be avoided. Although data are lacking to show clear benefits of adding mineralocorticoid, fludrocortisone at a dose of 0.1 to 0.2 mg twice a day can be used once the diagnosis has been made to limit excessive sodium excretion by the kidneys [14]. Alternatively, corticotropin has been used in similar cases to reduce the renal sodium excretion [10]. However, mineralocorticoids should be used sparingly in patients with excessive natriuresis despite salt and fluid replacement as they may have contributed to gradient medullary washout in some cases [9–13, 15–19].

In the present case, the observed polyuria was initially caused by severely reduced sodium and free water renal reabsorption, where these excessive renal losses were further exacerbated by the considerable fluid replacement volume administered to maintain appropriate cerebral perfusion. Concomitant administration of milrinone, which is known to increase renal perfusion (and therefore, associated hyperfiltration), might have contributed to polyuria. Indeed, the increased tubular flow rate over several days contributed to the progressive reduction of medullary interstitial tonicity by inhibiting urea concentration, further decreasing the capacity to maintain physiologic medullary countercurrent exchange, an essential function for urine concentration in long-loop nephrons [8]. The capacity to normalize that cortico-medullary osmotic gradient was further affected by the low serum (and therefore urinary) urea level due to hyperfiltration and low protein intake. This phenomenon led to progressive medullary solute washout and, thus, an inability to concentrate urine by the distal tubules. In the absence of tissue urea measurement, the participation of this phenomenon of medullary washout remains hypothetical. However, similar explanations for sustained polyuria have been reported in infants with glioblastoma [20] as well as in animal models [21]. Although urea administration is mainly reserved for cases of SIADH, here, its administration was aimed to reconstitute the osmotic gradient along the cortico-medullary axis (Fig. 2) as shown by the gradual increase of urine osmolality confirming the normalization of urine concentration capacity (Fig. 1).

Based on this case, we believe that secondary medullary washout following compensated cerebral salt wasting should be considered a potential complication of subarachnoid hemorrhage. Fluid management was a significant challenge in this patient, and hypovolemia had to be avoided. DCI complicated the management of this case because the maintenance of a desired higher cerebral perfusion constantly required a positive fluid balance that also contributed to increase urinary flow and further progressed to severe medullary washout. Indeed, in other clinical settings, a negative fluid balance should have led to extracellular volume depletion, and prerenal physiology, leading to a progressive decline in glomerular and tubular flow, resulting in urea reabsorption and normalization of the medullary concentration gradient. However, the neurological condition with volume-dependent vasospasm did not allow us to wait until a physiological phenomenon occurs. Despite not been used here, thiazide diuretics might have been an option in refractory cases because of their paradoxical antidiuretic effect [22, 23]. Although not based on strong clinical data, agents that decrease the glomerular filtration rate by acting on afferent or efferent renal arterioles, such as renin–angiotensin–aldosterone system inhibitors or calcineurin inhibitors could have been an alternative to the use of high-dose NSAIDS.

Cerebral salt wasting can lead to serious consequences in intracranial pathologies, especially when cerebral perfusion pressure is compromised due to relative hypovolemia. The differential diagnosis of patients presenting with polyuria in the context of acute neurological impairment should always include this entity in clinical management. Medullary washout should be suspected and managed accordingly in cases of severe and sustained polyuria. Although complex, early recognition of this entity could help to maintain and eventually restore medullary concentration gradient while limiting excessive fluid replacement by instituting early adequate treatment based on pathophysiological concepts discussed earlier. Further research is required to determine the optimal approach for this rare entity.

## Data Availability

To ensure protection of the patient privacy, no data will be shared.

## Abbreviations

ADH: Antidiuretic hormone
ANP: Atrial natriuretic peptide
BNP: Brain natriuretic peptide
CSF: Cerebrospinal fluid
CSW: Cerebral salt wasting syndrome
CT: Computed tomography
DCI: Delayed cerebral ischemia
GCS: Glasgow Coma Score
NIHSS: National Institute of Health Stroke Scale
NSAID: Non-steroid anti-inflammatory drug
SAH: Subarachnoid hemorrhage
SIADH: Syndrome of inanappropriate secretion of antidiuretic hormone
